# A New Antiseptic

**Published:** 1879-09

**Authors:** 


A New Antiseptic.—A new antiseptic agent has appeared in Germany, which, if the statements regarding it are true, is one of the most important yet discovered. It is a double salt of borate of potassium and sodium, and is made by dissolving in water equal quantities of chloride of potassium, nitrate of sodium, and boracic acid, and evaporating to dryness after filtering. Its cost is about twenty-five cents a pound, and its use in foods, etc., does not in the least injuriously affect them, and gives no taste or smell to substances. It has been extensively employed already by butchers, sausage makers, tanners, etc.; but its most important use is at present in the manufacture of butter and cheese from sweet milk. When butter is made from sweet milk in the ordinary manner, the milk must be kept very cold; when the "preserving salt," as it is called in Germany, is used, the milk may be kept at ordinary temperature without souring; the remaining sweet milk may be worked up into a superior quality of cheese. If fifteen grains of the salt are added for each quart of milk, the latter will keep sweet for at least a week. Fresh meat, game, etc., may be preserved by dipping it into a solution of one pound of the salt in six pints of water. When the meat is intended to be kept for a long period it is rubbed in well with the powdered salt in the proportion of one and one-half drachm to each two pounds of meat. In twenty-four hours the impregnation is completed, and it only needs to be dried. A piece of meat prepared in this manner in January, 1877, was in perfectly good condition in January, 1879. For pickling the meat is prepared in the same manner, and then placed between layers of a mixture of two pounds of common salt, and one half pound preserving salt, and one-fourth pound of sugar. In this way the largest hams can be salted in four days. For preserving skins, from one half to two pounds are used, according to size. Eggs are placed for fifteen minutes in a solution of one ounce of the salt in a quart of water. To preserve beer, wine, etc., it is sufficient to rinse the bottles, previous to filing them with a solution of the salt in the proportion of one to ten, and adding to the beverage itself eight grains per quart. For fish, lobsters, oysters, fruit. and vegetables, the preparation has also been used with the best success.—*Boston Journal of Chemistry.*

